# Molecular cloning and biochemical characterization of indole-3-acetic acid methyltransferase from Japanese star anise (*Illicium anisatum*)

**DOI:** 10.5511/plantbiotechnology.23.1224a

**Published:** 2024-03-25

**Authors:** Takao Koeduka, Ako Nakabo, Ami Takata, Ryo Ikeda, Hideyuki Suzuki, Sakihito Kitajima, Shin-ichi Ozaki

**Affiliations:** 1 Graduate School of Sciences and Technology for Innovation, Yamaguchi University, Yamaguchi 753-8515, Japan; 2 Department of Research and Development, Kazusa DNA Research Institute, Chiba 292-0818, Japan; 3 Department of Applied Biology, Kyoto Institute of Technology, Kyoto 606-8585, Japan

**Keywords:** basal angiosperm, indole-3-acetic acid, Japanese star anise, methyltransferase, SABATH

## Abstract

SABATH proteins methylate the carboxyl groups or nitrogen atoms of small plant molecules and play important roles in many developmental processes and plant defense responses. Previous studies have shown that indole-3-acetic acid (IAA) carboxyl methyltransferase (IAMT), a member of the SABATH methyltransferase family, converts IAA into its methyl ester (Me-IAA). We used RNA-seq analysis to identify a putative *IAMT* gene, *IaIAMT*, in the ancient angiosperm *Illicium anisatum*. Functional characterization of the recombinant IaIAMT protein expressed in *Escherichia coli* showed the highest level of activity with IAA, whereas indole-3-propionic acid and indole-3-butyric acid were not used as substrates. The apparent *K_m_* value of IaIAMT using IAA as a substrate was determined to be 122 µM. Phylogenetic analysis and structural modeling of IaIAMT suggested that IaIAMT evolved independently from IAMTs isolated from other plant species, whereas strict substrate specificity toward IAA was conserved in *Illicium* species, as observed in other plants.

## Introduction

In many plant species, phytohormones function as important signaling molecules for plant growth and development and diverse responses to environmental stresses such as drought, herbivory, and pathogen attack. Chemical modifications of phytohormones can significantly impact their activities and play a critical role in homeostasis (Westfall et al. 2013). For example, methylation and demethylation are rapid switching reactions of the chemical properties of metabolites, and the methylated forms are generally as inactive as hormones (Yang et al. 2008). Previous studies have shown that indole-3-acetic acid (IAA), gibberellic acid (GA), and jasmonic acid (JA) undergo methylation of their free carboxyl groups (Seo et al. 2001; Zhang et al. 2020; Zhao et al. 2008). In *Arabidopsis*, the overexpression of methyltransferases active on IAA leads to a dramatic leaf curvature phenotype (Qin et al. 2005). Similarly, in planta, overexpression of methyltransferases that convert GA to its methyl ester results in a dwarf phenotype in plants with low GA levels in vivo (Varbanova et al. 2007).

Enzymes that catalyze the transfer of a methyl group from *S*-adenosyl-L-methionine (SAM) to a carboxyl group of phytohormones have been identified in various plants and are members of the plant protein family SABATH. Salicylic acid methyltransferase (SAMT), indole-3-acetic acid methyltransferase (IAMT), jasmonic acid methyltransferase (JMT), and gibberellic acid methyltransferase (GAMT), which catalyze the methylation of the carboxylic acid of phytohormones such as SA, IAA, JA, and GA, respectively, are members of the SABATH family (Koeduka et al. 2020; Zubieta et al. 2003). Other SABATH methyltransferases include those that methylate benzoic acid, farnesoic acid, nicotinic acid, cinnamic/*p*-coumaric acid, carlactonoic acid, and nitrogen methyltransferases involved in caffeine biosynthesis (Effmert et al. 2005; Kapteyn et al. 2007; Kato et al. 2000; Mashiguchi et al. 2022; Wakabayashi et al. 2021; Yang et al. 2006). Since a wide range of compounds are used as substrates for SABATH methyltransferases, the SABATH family is considered a useful model for understanding the functional evolution and reaction mechanisms underlying substrate discrimination of a protein family.

Numerous SABATH enzymes in plants have been functionally characterized for their ability to methylate phytohormones. Recent studies of SABATH family have identified IAMTs in *Arabidopsis*, rice, lotus, black cottonwood, and white spruce (Goto et al. 2022; Zhao et al. 2007, 2008, 2009). Although SABATH enzymes with SAMT activity have been isolated from the liverwort *Conocephalum* and basal angiosperms, including *Magnolia*, *Annona*, and *Sassafras*, there have been no reports of the SABATH enzymes with IAA-methylating activity in basal angiosperms and bryophyte, including *Marchantia polymorpha* and *Physcomitrella patens*, for which whole-genome sequences are available (Dubs et al. 2022; Zhang et al. 2019). The *P. patens* genome contains four *SABATH* genes, none exhibiting phytohormone activity, including IAA (Zhao et al. 2012). Therefore, further studies are required to understand the functional evolution and diversification of SABATH activity during land-plant evolution.

This study aimed to gain further insights into the functional evolution of SABATH proteins that catalyze phytohormone methylation. To achieve this, we characterized the SABATH family in Japanese star anise (*Illicium anisatum*), a basal angiosperm that belongs to the Schisandraceae family and is native to Japan. We isolated and functionally characterized *Illicium* SABATH proteins (IaIAMT) responsible for methylating the carboxyl group of IAA with strict substrate specificity. Finally, we generated a homology model of IaIAMT to understand the molecular mechanisms underlying its strict substrate specificity.

## Materials and methods

### Plant materials and chemicals

*Illicium anisatum* plants were obtained from the Nihon Kaki Garden Center (Saitama, Japan) and grown outside the Yamaguchi University campus. After collection, the young leaves were immediately used for RNA extraction. All the chemicals used in this study were purchased from Sigma-Aldrich (St. Louis, MO, USA), Tokyo Kasei (Tokyo, Japan), or Fujifilm Wako (Osaka, Japan).

### RNA-seq analysis

RNA-seq was performed as previously described (Amano et al. 2018), with minor modifications. Briefly, total RNA was extracted from *I. anisatum* leaves using a combination of the CTAB method and Plant Total RNA Extraction Mini Kit (Favorgen Biotech, Ping-Tung, Taiwan). RNA quality was evaluated using BioAnalyzer 2100 (Agilent Technologies, USA). A 10 µg aliquot of total RNA was used to construct a cDNA library using the Illumina TruSeq Prep Kit v2, according to the manufacturer’s protocol (Illumina, USA). The resulting cDNA library was sequenced using MiSeq (Illumina) in high-output mode with 150 bp paired-end reads. Reads were assembled using the CLC Genomics Workbench version 7.0.4 (CLC Bio, Japan) with the following parameters: a minimum contig length of 200 bp and scaffolding after adaptor sequences and low-quality reads were removed. All raw read sequences are available in the NCBI Sequence Read Archive under the accession number DRA017248.

### Sequence and phylogenetic analysis of putative SABATH proteins from Japanese star anise

To identify putative *SABATH* genes, AtJMT, AtBSMT, AtIAMT1, and CbSAMT protein sequences from *A. thaliana* and *Clarkia breweri* were used to search the RNA-Seq database from *I. anisatum* leaves as protein queries. For phylogenetic analysis, multiple protein sequence alignments, including IaSABATHs, were constructed using the MAFFT program (version 7) (Katoh and Standley 2013), and a neighbor-joining tree was constructed using the MEGA program (version 6) (Tamura et al. 2013) with 1000 bootstrap replicates. Details of the sequences used to build the trees are listed in Supplementary Table S1.

### Protein expression of IaSABATHs in *Escherichia coli*

The full-length *IaSABATH* cDNAs was obtained by PCR amplification with gene-specific primers (Supplementary Table S2) using a cDNA template synthesized from *I. anisatum* leaf RNA. PCR products were separated on a 1.2% agarose gel and purified using a GEL/PCR Purification Mini Kit (Favorgen Biotech). cDNA was cloned into the pGEM-T easy TA-cloning vector (Promega, Madison, WI, USA) and confirmed by sequencing. For protein expression in *E. coli*, full-length *IaSABATH* genes were transferred to the expression vector pCold-maltose-binding protein (MBP), which is a modified pCold-I vector (Takara Bio Inc., Shiga, Japan), to yield an N-terminal MBP-fusion protein. The resulting plasmids were transfected into *E. coli* BL21(DE3) competent cells. Recombinant proteins were expressed in the presence of isopropyl β-D-1-thiogalactopyranoside (0.5 mM) for 24 h at 16°C. IaSABATH fusion proteins expressed with MBP were purified using gelatinized corn starch, as described by Kobashigawa et al. (2021). The protein concentration was determined using the Bio-Rad Protein Assay Dye Reagent with BSA as a standard.

### Enzyme assays and kinetic analysis

Enzyme assays for IaIAMT and related IaSABATHs were performed using radiochemical methods and purified recombinant MBP fusion proteins, as previously described (Kobashigawa et al. 2021; Koeduka et al. 2016). Briefly, 200 mM (final concentration) sodium phosphate (pH 7.0), 380 ng of purified proteins, 1 mM substrate, and 2 µM [Me-^14^C]-SAM (58.0 mCi mmol^−1^; PerkinElmer, Boston, MA) were combined in 50 µl reactions and incubated at 25°C for 10 min. The reactions were stopped by the addition of 200 µl ethyl acetate, the samples were centrifuged for 1 min at 12,000×g, and the radioactivity of the organic phase was measured using a scintillation counter (AccuFLEX LSC-7200; Hitachi Aloka Medical, Ltd., Tokyo, Japan). For product identification, a reaction contained 25 µg of purified proteins, 1 mM substrate (IAA), 1 mM SAM, and 100 mM sodium phosphate (pH 7.0) in a final volume of 1 ml, and was incubated for 7 h at 25°C. The enzymatic products were extracted with 2 ml ethyl acetate and analyzed by gas chromatography-mass spectrometry (GC-MS). GC-MS analysis was performed with a GC-MS (QP-2010 Plus, Shimadzu, Kyoto, Japan) equipped with a DB-5ms column (30 m length×0.25 mm diameter×0.25 µm film thickness, Agilent Technologies, Santa Clara, CA, USA). The initial oven temperature was 100°C, held for 1 min, ramped up at 10°C min^−1^ to 170°C, and subsequently increased by 20°C min^−1^ to 240°C, and held for 6 min. The temperature of the ion source and interface was 240°C with a continuous scan ranging *m*/*z* 60–300. For kinetic analysis, various amounts of IAA (0–400 µM) and 950 ng of purified protein were used in a 50 µl reaction, which was incubated for 10 min at 25°C, as previously described (Koeduka et al. 2020). All reactions were performed in triplicates.

### Construction of homology-model and molecular docking

An open-form model of IaIAMT was generated using the Swiss-model (https://swissmodel.expasy.org/interactive), with the crystal structure of AtIAMT (PDB code: 3B5I) complexed with *S*-adenosyl homocysteine (SAH) as a template. To construct the closed-form model, CbSAMT (PDB code: 1M6E), a ternary complex crystal structure containing SAH and SA, was used as a template. The generated model structures were superimposed on templates to locate SAH in the active sites, and those models in complexed with SAH were used as reference proteins for molecular docking. The structures of the ligands and methyl acceptors, such as IAA, IPA, and IBA, were obtained from PubChem and saved in .pdb format using PyMol. Both the reference proteins and ligands were loaded onto the Python Molecular Viewer and converted to .pdbqt format. The grid box was centered at the active site, and the docking simulation was performed using AutoDock Vina. Plausible binding orientations for the methyl acceptors were shown using PyMol. Molecular docking of IPA and IBA was also performed using the models in the absence of SAH as a reference protein.

## Results and discussion

### Isolation and sequence analysis of *SABATH* genes from Japanese star anise

BLAST analysis by comparison with known SABATH proteins in the RNA-seq database of *I. anisatum* leaves revealed four SABATH-like sequences with more than 40% identity. These four candidates were designated IaSABATH1 (DDBJ accession number LC762307), IaSABATH2 (DDBJ accession number LC762308), IaSABATH3 (DDBJ accession number LC762309), and IaSABATH4 (DDBJ accession number LC762310). The sizes of these IaSABATHs ranged from 358 to 409 amino acids, in length and the sequence identities among the IaSABATHs ranged from 29% to 47%. The IaSABATH1 protein sequence exhibited 63% sequence identity with *Arabidopsis* IAMT (AtIAMT) and IaSABATH2 exhibited 67% sequence identity with *Citrus clementina* GAMT (CcGAMT1). In addition, the sequence identities of IaSABATH3 and IaSABATH4 with known carboxyl methyltransferases found in higher plants were 50% and 48%, respectively, which are similar to *Clarkia* SAMT (CbSAMT) (Supplementary Table S3).

### Biochemical properties of IaSABATHs from Japanese star anise

**Figure 1:**
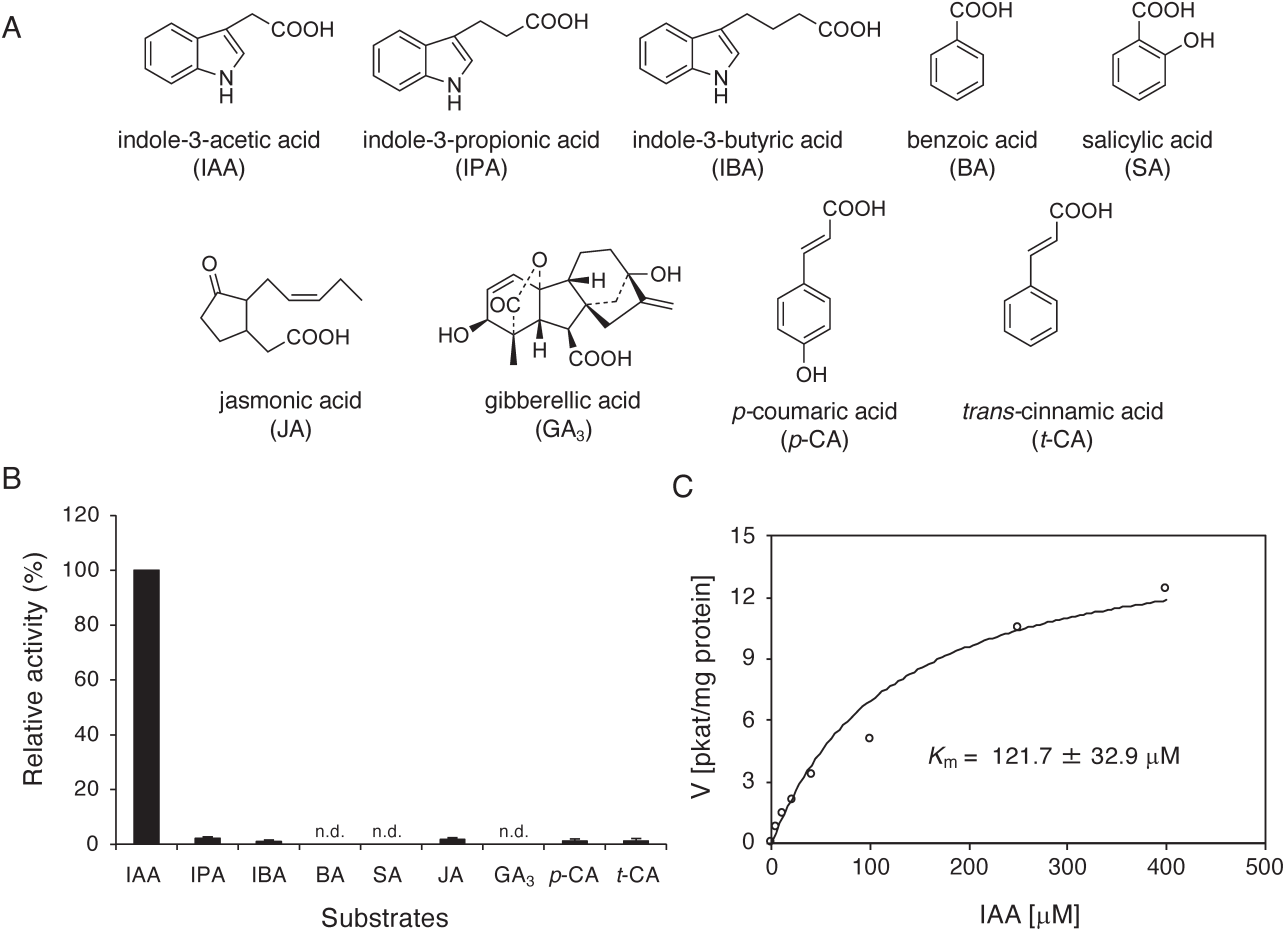
Figure 1. Enzymatic activity of *I. anisatum* IAA carboxyl methyltransferase. (A) Substrates used to measure the relative activity of IaIAMT. (B) Relative activities of IaIAMT against the activity of indole-3-acetic acid. The means are shown as SE (*n*=3). (C) Kinetic analysis of IaIAMT. The kinetic parameters of IaIAMT are shown (inset).

**Figure 2:**
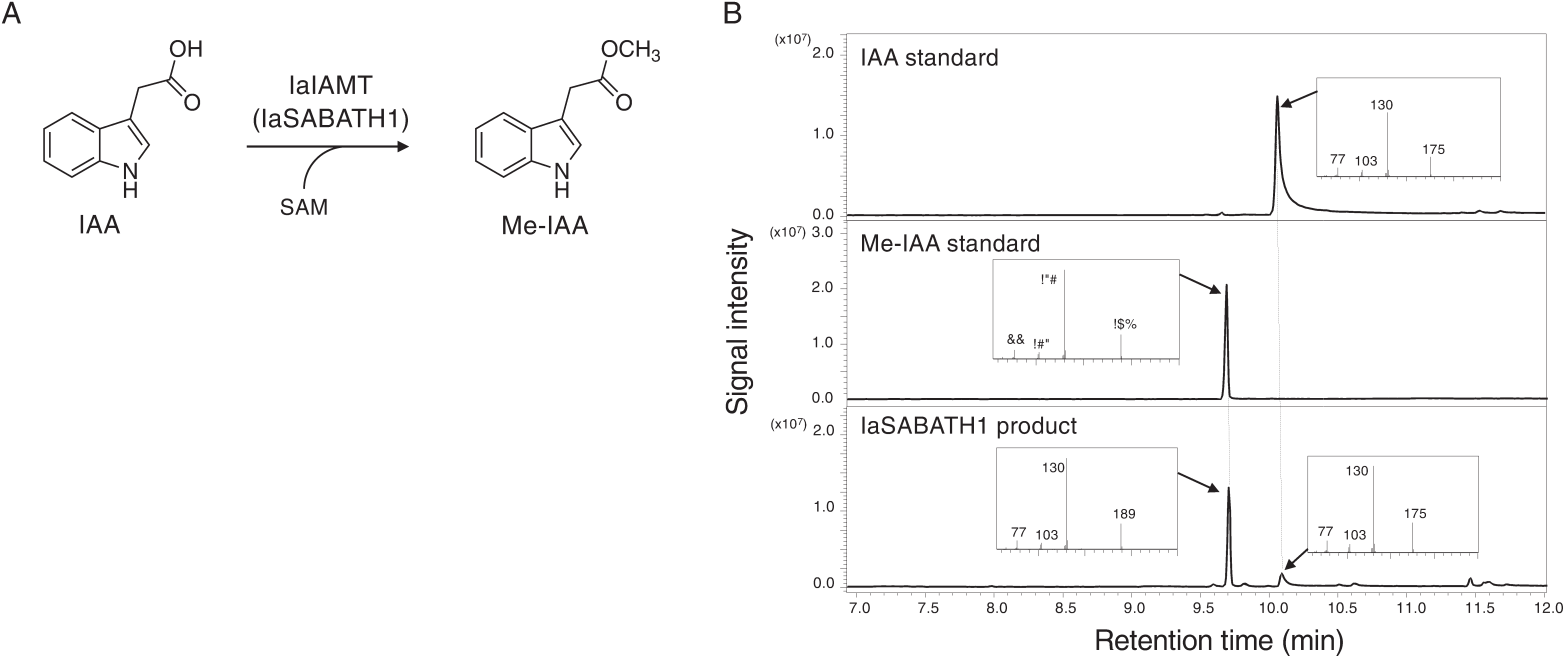
Figure 2. IaIAMT from *I. anisatum* encodes a functional indole-3-acetic acid carboxyl methyltransferase in vitro. (A) IaIAMT catalyzes the methylation of indole-3-acetic acid (IAA) to produce methyl indole-3-acetic acid (Me-IAA). (B) GC-MS analysis of the products formed in vitro by the enzymatic activities of purified IaIAMT using indole-3-acetic acid as substrates.

To test the in vitro enzymatic activities, the four IaSABATHs recombinant proteins were heterologously expressed in *E. coli* as His-tagged or MBP-fusion proteins. Cell lysates expressing individual IaSABATH proteins were assayed using several putative substrates (Figure 1A). Although IaSABATH2, IaSABATH3, and IaSABATH4 showed no activity against any of the nine substrates tested, IaSABATH1 showed the highest level of IAA-specific activity and was thus renamed IaIAMT (Figure 1B). The recombinant IaIAMT fusion protein with MBP was purified using gelatinized starch (Supplementary Figure S1), and IAA was converted into its methyl ester in the presence of SAM as a methyl donor. The enzymatic products were identified as methyl indole-3-acetic acid (Me-IAA) by comparison with an authentic Me-IAA standard using GC-MS (Figure 2). However, IaIAMT exhibited subtle activity toward JA, *p*-CA, and *t*-CA (1.0–2.1% relative activity toward IAA). In contrast, the enzyme displayed negligible activity against BA, SA, and GA_3_ (Figure 1B). High specificity of IaIAMT toward IAA substrates has also been observed in IAMTs from *Arabidopsis* (AtIAMT), black cottonwood (PaIAMT), and white spruce (PgIAMT) (Zhao et al. 2007, 2008, 2009). Interestingly, although IaIAMT was highly active toward IAA, we did not detect substantial methyltransferase activity for IPA and IBA, as shown in Figure 1B. This suggests that IaSABATH1 can discriminate between chemical structures of IAA derivatives with different side chain lengths. Further kinetic assay of IaIAMT demonstrated that the apparent *K_m_* value of IaIAMT for IAA was 121.7±32.9 µM (Figure 1C). The *K_m_* value was 5–9-fold higher than that observed for other IAMTs found in *Arabidopsis*, rice, poplar, and white spruce (Qin et al. 2005; Zhao et al. 2007, 2008, 2009), suggesting that IAA homeostasis regulated by methyltransferases in basal angiosperms or Japanese star anise may be different from that of other plants and angiosperms. However, further identification of IAMT proteins in different basal angiosperms is required to prove this hypothesis.

### Structural models of IaIAMT

**Figure 3:**
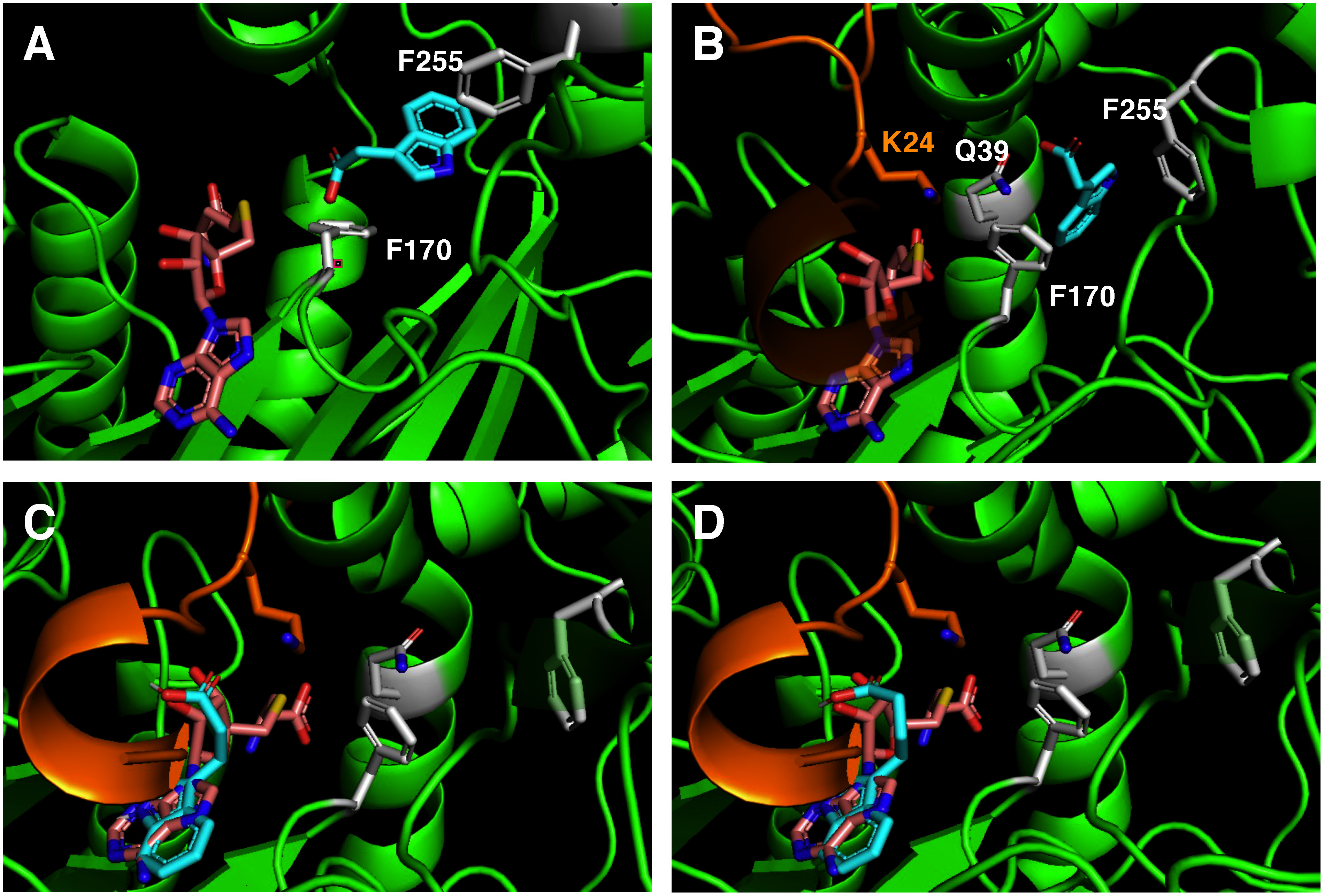
Figure 3. Views of the active sites of IaIAMT models. (A) An open-form IaIAMT model constructed with the crystal structure of *Arabidopsis* IAMT (PDB code: 3B5I) as a template. IAA (cyan) was docked into the active site of the model in complex with SAH (wheat). The residues (Phe-170 and Phe-255 in IaIAMT) important for substrate positioning are shown in stick style. (B) A closed-form IaIAMT model constructed with the crystal structure of *Clarkia* SAMT (PDB code: 1M6E) as a template. IAA (cyan) was docked into the active site of the model in complex with SAH (wheat). The residues (Lys-24, Gln-39, Phe-170, and Phe-255 in IaIAMT) important for substrate positioning are shown in stick style. The N-terminal domain in IaIAMT is shown in orange. A predicted binding site for (C) IPA (cyan) and (D) IBA (cyan) in the active site of a closed-form IaIAMT model, suggesting that IPA and IBA may bind in the SAH (wheat) site.

Structural models of IaIAMT were generated to elucidate its substrate specificity for IAA, IPA, and IBA. The active site of the IaIAMT model was superimposed on the X-ray structures of CbSAMT (closed-form model) and AtIAMT (open-form model) (Figure 3A, B). The simulation suggested that the IAA substrate was docked into the active site such that its carboxyl moiety was located at a suitable position for accepting the methyl group from SAM (Figure 3). As previously reported for AtIAMT (Zhao et al. 2008), the N-terminal domains of IaIAMT also form a mobile active site capping loop and are involved in dimer formation, part of which is close to the carboxyl moiety of the IAA substrate, orienting the methyl acceptor close to the SAM (Figure 3B). The active sites of both the open- and closed-form models accommodated IAA in reactive configurations. The docking structure in the closed-form model suggested that the active-site residues (Lys-24, Gln-39, Phe-170, and Phe-255 in IaIAMT; Supplementary Figure S2) likely interacted with IAA and were responsible for determining the position of the IAA substrate at the active site (Figure 3B). In contrast to the docking model with IAA, when IPA and IBA were docked into the active site of the closed-form model, the active sites were unable to accommodate bulky acceptors and SAM for the methyl transfer reaction (data not shown). Further simulation studies implied that IPA and IBA may exist at the SAM-binding site (Figure 3C, D). This finding is consistent with the strict specificity of the IaIAMT for IAA.

### Phylogenetic tree analysis of IaIAMT with other SABATH proteins

**Figure 4:**
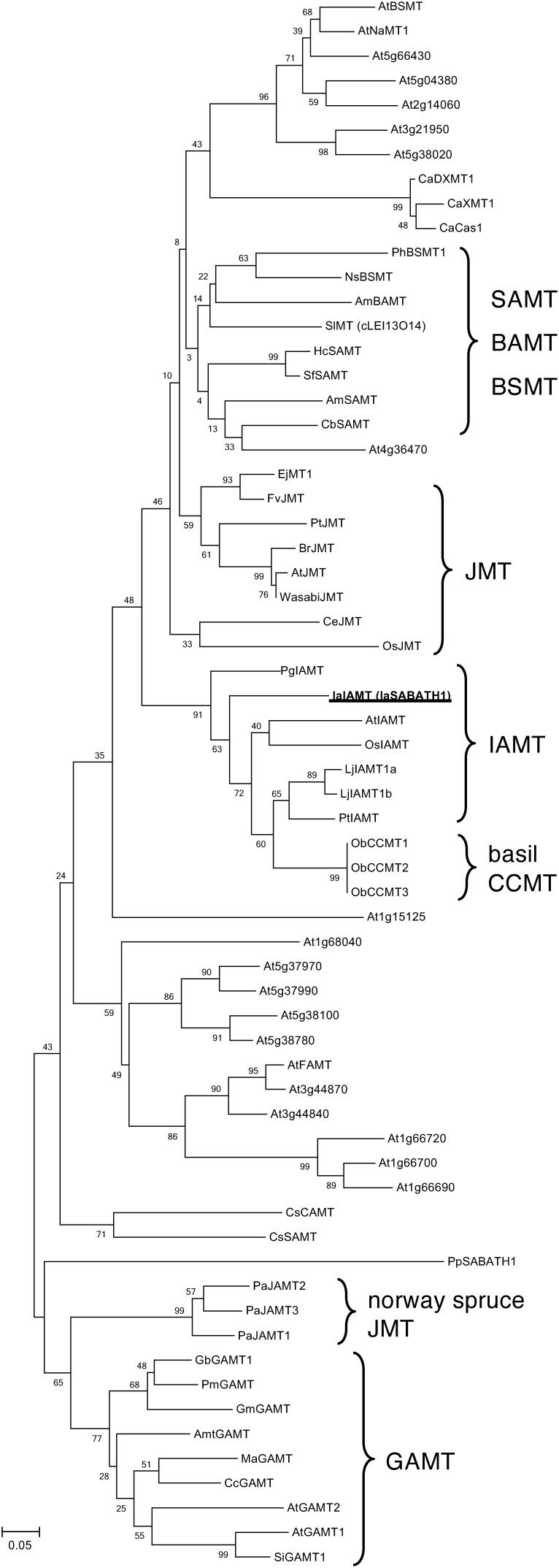
Figure 4. Phylogenetic relationship of *I. anisatum* IAA carboxyl methyltransferase with other related SABATHs from different plant species. Phylogenetic analysis was performed using the neighbor-joining method in the MEGA6 software. Scale bar represents 0.05 amino acid substitutions per site. The IaIAMT characterized in this study is highlighted in bold.

A phylogenetic tree was constructed using the Japanese star anise IaIAMT, all SABATH proteins from *Arabidopsis*, and functionally characterized SABATH proteins from higher plants to understand the evolutionary relationship between IaIAMT and other SABATHs (Figure 4). IaIAMT was evolutionarily closely related to IAMTs from other plant species, *Arabidopsis* (AtIAMT), rice (OsIAMT), white spruce (PgIAMT), *Lotus japonicus* (LjIAMT1a and LjIAMT1b), and poplar (PtIAMT) with basil cinnamate/*p*-coumarate carboxyl methyltransferases (ObCCMT1, ObCCMT2, and ObCCMT3). However, IaIAMT is located separately from other IAMTs isolated from eudicots and monocots in the phylogenetic tree (Figure 4). Therefore, it is likely that IAMT proteins belonging to basal angiosperms evolved into a lineage distinct from that of seed plants during plant evolution.

To the best of our knowledge, this is the first study to identify the IAMT in basal angiosperms. It would be interesting to determine the presence of IAMT in different basal angiosperms and whether IAMT proteins fall into the same clade as IaIAMT. Overall, our results provide biochemical evidence that deepens the previous knowledge of the high specificity of the plant SABATH protein family toward IAA.

## Description of Supplementary Files

Supplementary Table S1. Accession numbers of sequences used in phylogenetic trees.

Supplementary Table S2. Oligonucleotide sequences used for the cloning of full length *IaSABATH* genes.

Supplementary Table S3. Sequence analysis of IaSABATH proteins used in this study.

Supplementary Figure S1. SDS-PAGE analysis of the purification of recombinant IaIAMT.

Supplementary Figure S2. Protein sequence alignment of *I. anisatum* IAMT and the related SABATHs from other plant species.
