# Supplementary material for: Molecular cloning and biochemical characterization of indole-3-acetic acid methyltransferase from Japanese star anise (Illicium anisatum)
Source: Plant Biotechnol (Tokyo). 2024 Mar 25;41(1):65–70. doi: 10.5511/plantbiotechnology.23.1224a (PMC11500593; doi:10.5511/plantbiotechnology.23.1224a)
Supplement: Supplementary Data [file plantbiotechnology-41-1-23.1224a-s001.pdf]

Supplementary Table S1

Accession numbers of sequences used in phylogenetic trees

Gene name	Binomial name	Accession number
AmtGAMT	*Amborella trichopoda*	MW149497
AtIAMT (At5g55250)	*Arabidopsis thaliana*	NP_200336
AtGAMT1 (At4g26420)		F4JUY5
AtGAMT2 (At5g56300)		Q5XF78
AtJMT (At1g19640)		Q9AR07
AtBSMT (At3g11480)		Q6XMI3
At1g15125		A0A1P8APB9
At1g66700		Q9C9M3-1
At1g66720		Q9C9M4-1
At1g66690		Q9C9M2-1
At1g68040		Q9C9W8-1
At2g14060		F4IFF4-1
At3g21950		Q9LRL5
At3g44840		Q9FYC6-1
At3g44870		Q9FYC3-1
AtFAMT (At3g44860)		Q9FYC4-1
At4g36470		O23234-1
At5g04380		F4JW90-1
AtNaMT1 (At5g04370)		Q0WL44-1
At5g37970		Q9FKD0-1
At5g37990		Q9FKC8-1
At5g38020		Q84MB1-1
At5g38100		Q9LS10-1
At5g38780		Q9FKR0-1
At5g66430		Q9FJZ6-1
AmBAMT	*Antirrhinum majus*	Q9FYZ9
AmSAMT		Q8H6N2
BrJMT	*Brassica rapa*	XP_033133906
CaCas1	*Coffea arabica*	AB086414
CaDXMT1		AB084125
CaXMT1		AB048793
CbSAMT	*Clarkia breweri*	Q9SPV4
CcGAMT	*Citrus clementina*	MW149509
CeJMT	*Cymbidium ensifolium*	JQ360571
CsCAMT	*Conocephalum salebrosum*	MK673137
CsSAMT		MK673138
EjMT1	*Eriobotrya japonica*	LC127197
EujJMT	*Eutrema japonicum*	LC543983
FvJMT	*Fragaria vesca*	XP_004291853
GbGAMT1	*Ginkgo biloba*	MW149493
GmGAMT	*Gnetum montanum*	MW149492
HcSAMT	*Hoya carnosa*	CAI05934
IaIAMT (IaSABATH1)	*Illicium anisatum*	LC762307
LjIAMT1a	*Lotus japonicus*	Lj2g3v3222870
LjIAMT1b		Lj6g3v0819010
MaGAMT	*Musa acuminata*	MW149500
NsBSMT	*Nicotiana sylvestris*	ACZ55223
ObCCMT1	*Ocimum basilicum*	EU033968
ObCCMT2		EU033969
ObCCMT3		EU033970
OsIAMT	*Oryza sativa*	Q0J998
OsJMT		XP_015639512
PaJAMT1	*Picea abies*	MA_128083g0020
PaJAMT2		MA_9561g0010
PaJAMT3		MA_10259819g0010
PgIAMT	*Picea glauca*	Zhao et al. (2009) Tree Physiol
PhBSMT1	*Petunia hybrida*	AAO45012
PmGAMT	*Pseudotsuga menziesii*	MW149495
PpSABATH1	*Physcomitrella patens*	Zhao et al. (2012) *Phytochemistry*
PtIAMT	*Populus trichocarpa*	XP_002298843
PtJMT		AGR50489
SfSAMT	*Stephanotis floribunda*	CAC33768
SiGAMT	*Sisymbrium irio*	MW149509
SlMT (cLEI13O14)	*Solanum lycopersicum*	AW650581

Supplementary Table S2

Oligonucleotide sequences used for the cloning of full length IaSABATH genes

Genes	Direction	Primer sequence (5'→3')
IaSABATH1(*IaIAMT*)		
IaSABATH1/F	Forward	AACATATGGCTATGGCTATGAAAGAAG (underline indicates *Nde*I site)
IaSABATH1/R	Reverse	TTGTCGACTTACTTAAAAAGGGAAAGGGAG (underline indicates *Sal*I site)
IaSABATH2		
IaSABATH2/F	Forward	AACATATGGAACCCACCATGGAATCT (underline indicates *Nde*I site)
IaSABATH2/R	Reverse	TTGTCGACTCATATCCTAAAGGCACAAACA (underline indicates *Sal*I site)
IaSABATH3		
IaSABATH3/F	Forward	AACATATGGATGTGAAGAACGCTCTC (underline indicates *Nde*I site)
IaSABATH3/R	Reverse	TTGTCGACTTACCCTTTTCTTTTGAGGACT (underline indicates *Sal*I site)
IaSABATH4		
IaSABATH4/F	Forward	AACTCGAGATGGACATAAACCAGGTTCTAC (underline indicates *Xho*I site)
IaSABATH4/R	Reverse	TTGTCGACTCATTCTTTCTTTGTCATGGATAT (underline indicates *Sal*I site)

Supplementary Table S3

Sequence analysis of IaSABATH proteins used in this study

Protein name	Protein size (amino acids)	Calculated molecular weight (kDa)	Highest similar protein	
IaSABATH1 (IaIAMT)	387	42.8	AtIAMT	63%
IaSABATH2	409	46.6	CcGAMT	67%
IaSABATH3	358	34.4	CbSAMT	50%
IaSABATH4	370	41.1	CbSAMT	48%

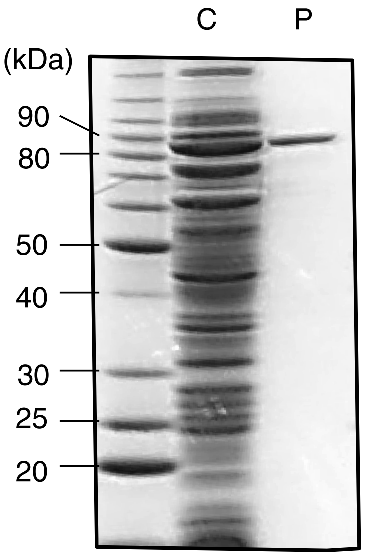

Supplementary Figure S1. SDS-PAGE analysis of the purification of recombinant IaIAMT.

IaIAMT was expressed as an MBP-fused protein and purified by using gelatinized starch. C: crude enzyme; P: purified enzyme.

```
                                          Lys-24      Gln-39
                                          &
                                          ▲      & ▲                                     &
IaIAMT      1  ----------------MAMAMKEDSMLLPQKKLEKLFSMKGGKGETSYAKNSQAQAKHARSMLHLLEGALDGMKLPSPESL--FTVADLGCSSGENTLFIIKAIVKHMAKRYKAAG-TNP
AtIAMT      1  ------------------MGSKGDNVAVCNMKLERLLSMKGGKGQDSYANNSQAQAMHARSMLHLLEETLENVHLNSSASPPPFTAVDLGCSSGANTVHIIDFIVKHISKRFDAAG-IDP
LjIAMT1a    1  ---------------------MGDNVVVSKMELERLLSMKGGKGEASYANNSQAQAIHAKSMLHLLKETLDRVKLGDADTP--FVVVDLGCSCGSNTIKVVDVIIKHIIKRYESLGCFDL
LjIAMT1b    1  ------------------MAHVGDNVVASNMELERLLSMKGGKGEASYANNSQAQAMHAKSMLHLLKEALDGVQLHATDIP--FVVVDLGCSCGDNTINVVDVIIKHIIKRYEALG-LHP
PtIAMT      1  ------------------MAPKGDNVVVSSMKLEKLLCMKGGKGEASYANNSQAQALHARSMLHLLEETLDRVHLNSPEFP--FQVADLGCSSGNNTIHIIDVIIKHMIKRFESSG-LEP
OsIAMT      1  -------MTMAMASMKGENVTVSAAAAPRMKKLASMLCMKGGNGDGSYLNNSQAQALHARRMLHFLEETLDAMMERSSSDK-LFTAADLGCSCGSNSLFIVDVIVRRVSEAYESRG-RDA
PgIAMT      1  MAFNFKTVCMAFSPAAATSIPADSPLTLMTRKLERLFSMQGGKGETSYAKNSQGQAKHLRSLRPLLEEALDAMDLPLSEEL--FTIADLGCSSGQNTLVIANHIVNHFANKYKLES-LNL
CbSAMT      1  -----------------------------MDVRQVLHMKGGAGENSYAMNSFIQRQVISITKPITEAAITALYSGDTVTTR-LAIADLGCSSGPNALFAVTELIKTVEELRKKMGRENS
                                         .:  :: *:** *: **  **  *         : : ::  :    .     :  .*****.* *::     ::. . :  .  .  .

                                                                                             Phe-170
                    &&                                              &&                       #  ▲
IaIAMT    102  PEFQAFFSDLPSNDFNTLFQLLP-QLPGN-GNLEHCLTSDE-----TRCYYAAGVPGSFYGRLFPSETINIFYSAFSLQWLSQVPESVMERCSPAYNSGRVFLHGASELTASAYRKQFQV
AtIAMT    102  PEFTAFFSDLPSNDFNTLFQLLP-PLVSN-TCMEECLAADG-----NRSYFVAGVPGSFYRRLFPARTIDFFHSAFSLHWLSQVPESVTDRRSAAYNRGRVFIHGAGEKTTTAYKRQFQA
LjIAMT1a   98  PEFSAFFSDLPSNDFNTLFQLLP-PLANYGVSMEECLAANN-----HRSYFAAGVPGSFYRRLFPAKSIDVFHSAFSLHWLSQVPESVLDKRSSAYNKGRVFIHGADESTTSAYKKQFQT
LjIAMT1b  100  PEFSAFFSDLPSNDFNTLFQLLP-PLANYGVSMEECLAANN-----HRSYFAAGVPGSFYRRLFPAKSVHIFHSAFSLHWLSQLPESVVDKRSRAYNKGRVFIHGASEMTANAYKKQFQT
PtIAMT    100  PEFSAFFADLPSNDFNTLFQLLP-PPANYGGSMEECLAASG-----HRNYFAAGVPGSFHRRLFPARSIDVFHSAFSLHWLSQVPECVLDKRSAAYNKGRVFIHNASESTTNAYKKQFQT
OsIAMT    112  PEFQVFFSDLPSNDFNTLFQLLPPLLAPVAGSLEECLAAGEGAATATRPYHAAGVPGTFYGRLFPGESIDVFTSTFSLHWLSQVPEEVGDSASPAYNGGRVFVHRATEAVAAAYKRQFQA
PgIAMT    118  PEFQVFFSDLPSNDFNTLFQLLPPIQETGAGVGVDCVNKEP------RAYHVAGVPGSFYKRLFPRNTINVVHSSFSLHWLSQVPETVQDPRSPAWNNGRVFIHKGSQLTANAYRTQFKN
CbSAMT     90  PEYQIFLNDLPGNDFNAIFRSLP-----------IENDVDG-------VCFINGVPGSFYGRLFPRNTLHFIHSSYSLMWLSQVPIGIESNKGNIYMAN-----TCPQSVLNAYYKQFQE
               **:  *: ***.****::*: **                                .  ****:*: **** .::... *::** ****:*  : .  .  :  .        : .  **  **:

                                                     Phe-255
                                     #             ###
IaIAMT    215  DFSGFLRCRSRELKKDGCMFLAFVGRTSQTPETTKVPGVLFGTHFQEAWNDLVLEGLLEAEKRDSFNIPVYAPSLQDFREVVEADGSFHINKLEIFRGGCALVVSHPN---DPSEVGRAF
AtIAMT    215  DLAEFLRARAAEVKRGGAMFLVCLGRTSVDPTDQGGAGLLFGTHFQDAWDDLVREGLVAAEKRDGFNIPVYAPSLQDFKEVVDANGSFAIDKLVVYKGGSPLVVNEPD---DASEVGRAF
LjIAMT1a  212  DLASFLSSRSVEMKRGGSMFLVCLGRTSVDPTDQGGAGLLFGTHFQDAWDDLVQEGLISREKRDNFNIPVYAPSLQDFKEVVEADGSFAINKLEVFKGGSPLVVNQPD---DASEVGRAL
LjIAMT1b  214  DLAGFLSSRSVEMKRGGSMFLVCLGRTSVDPTDQGGAGLLFGTHFQDAWDDLVQEGLISNEKRDNFNIPVYAPSLQDFKEVVEANGSFTINKLEVFKGGSPLVVNQPD---DANEVGRA-
PtIAMT    214  DLAGFLSARSQEMKSGGSMFLVCLGRTSADPTDQGGAGLLFGTHFQDAWDDLVQEGLITSEKRDNFNIPVYAPSLQDFKEVVEANGSFTIDKLEVFKGGSPLVVNHPD---NEAEVSRAM
OsIAMT    232  DLARFLRSRAREMKRGGAMFLACLGRSSGDPADQGGAGLLFGTHFQDAWDDLVQEGVVEGEKRDSFNIPVYAPSLQEFRDVVRADGAFAIDRLELVRGGSPLVVDRPD---DAAEVGRAM
PgIAMT    232  DFESFLRARSEEMKSGGCMFLVCLGRTFSDPTNQGGGGILFGTHFENAWNDLVDEGLIEAESRDTFNVPVFAPTVGEFRGVVEKENSFEIKKVDIFRGGSPLVVQCAE---DRQELGRAF
CbSAMT    187  DHALFLRCRAQEVVPGGRMVLTILGRRSED--RASTECCLIWQLLAMALNQMVSEGLIEEEKMDKFNIPQYTPSPTEVEAEILKEGSFLIDHIEASEIYWSSCTKDGDGGGSVEEEGYNV
               *   ** .*: *:  .* *.*. :**               *:   :  * :::* **::  *. * **:* ::*:  :..  :  :.:* *.::   .   .  ..  :   . * .

                                                         #
IaIAMT    335  TNSCRSVCGVLVDAHIGEQLSQVLFSRLESRATNDAKELLDQLHLSHIVASLSLFK-
AtIAMT    335  ASSCRSVAGVLVEAHIGEELSNKLFSRVESRATSHAKDVLVNLQFFHIVASLSFT--
LjIAMT1a  329  ANSCRAVCGVLVDAHIGDKHSEELFLRVEQRATNQAKELLEKLQFFHIVASLSLVQ-
LjIAMT1b       ---------------------------------------------------------
PtIAMT    334  ANSCRSVAGVLVDAHIGDGLSEELFLRVEHRAKSHAKELLEKLQFFHIVASLSFA--
OsIAMT    352  ANSCKAVAGVLVDAHIGERRGAQLFERLERRAARHARELVEKMHFFHVVCSLSLAP-
PgIAMT    352  TNSCRCVCGVLVDDHIGKDLSEKLFNRMEKRAIEHAEALLEDLEFFHVVAALTRK--
CbSAMT    305  ARCMRAVAEPLLLDHFGEAIIEDVFHRYKLLIIERMSK--EKTKFINVIVSLIRKSD
```

Supplementary Figure S2. Protein sequence alignment of *I. anisatum* IAMT and the related SABATHs from other plant species.

Gaps are represented by dashed lines (-). Asterisks (*), colon (:), and dots (.) indicate identical amino acid residues, strongly similar amino acid residues, and weakly similar amino acid residues between IaIAMT and the other SABATHs. Residues indicated with "▲" are residues that interact with the carboxyl moiety of the substrate. Residues indicated by "&" are important amino acids for the *S*-adenosyl-L-methionine (SAM)-binding residues. Residues indicated by "#" interact with the aromatic moiety of the substrate.
